# Neuronal transcription factor Brn-3a(l) is over expressed in high-grade ovarian carcinomas and tumor cells from ascites of patients with advanced-stage ovarian cancer

**DOI:** 10.1186/1757-2215-3-17

**Published:** 2010-07-29

**Authors:** Nuzhat Ahmed, Ardian Latifi, Clyde B Riley, Jock K Findlay, Michael A Quinn

**Affiliations:** 1 Women's Cancer Research Centre, Royal Women's Hospital, 20 Flemington Road, Parkville, Victoria 3052, Australia; 2 Department of Obstetrics and Gynaecology, University of Melbourne, 20 Flemington Road, Parkville, Victoria 3052, Australia; 3 Department of Surgery, University of Melbourne, Clinical Sciences Building, St. Vincent's Hospital, 29 Regent St, Fitzroy, Victoria 3065, Australia; 4 Prince Henry's Institute of Medical Research, Monash Medical Centre, 246 Clayton Road, Clayton, Victoria 3168, Australia

## Abstract

**Objectives:**

In view of the recent association of Brn-3 transcription factors with neuroblastomas, cervical, breast, and prostate cancers we examined the expression of Brn-3a(l) in normal ovaries and in different histological grades of ovarian tumors. The expression of Brn-3a(l) was also evaluated in normal ovarian and cancer cell lines and tumor cells isolated from the ascites of advanced-stage ovarian cancer patients.

**Methods:**

Normal ovaries, benign, borderline, grades 1, 2 and 3 ovarian tumors were analyzed by immunohistochemistry for Brn-3a(l) expression. A total of 46 ovarian specimens were included in the study. Immunofluorescence was used to investigate the expression of Brn-3a in normal ovarian and cancer cell lines. Brn-3a(l) expression was also evaluated by Western blot in tumor cells isolated from ascites of advanced-stage ovarian cancer patients and also in ovarian cancer cell lines.

**Results:**

Nearly 12% of normal and benign ovarian tissues and 57% of borderline ovarian tumors were positive for epithelial Brn-3a(l) expression. Stromal staining was higher and it constituted 40% of normal non-cancerous ovaries compared to 50 and 86% in benign and borderline tumors. On the other hand, 85-100% of grades 1, 2 & 3 ovarian tumors demonstrated nuclear and cytoplasmic Brn-3a(l) staining in the epithelium. Stromal staining in grades1, 2 and 3 tumors constituted 71-88% of total staining. Overall, immunoreactive Brn-3a was present in all grades of ovarian tumors. The extent of epithelial and stromal Brn-3a staining was significantly different between the normal and histological grades of tumors (epithelial-χ^2 ^= 41.01, df = 20, P = 0.004, stromal-χ^2 ^= 24.66. df = 15, P = 0.05). The extent of epithelial staining was significantly higher in grades 1 and 2 ovarian tumors compared to normal ovaries and benign ovarian tumors (p < 0.05). In parallel, stromal staining was significantly higher in grade 3 tumors compared to normal ovaries (p < 0.05). In addition, cytoplasmic and nuclear Brn-3a expression was evident in ovarian cancer cell lines while no such expression was observed in SV40 antigen immortalized normal ovarian cell lines.

**Conclusion:**

These data suggest that like other cancers, Brn-3a(l) expression is enhanced in ovarian tumors and its expression is consistent with its known role in inhibiting apoptosis and enhancing tumorigenesis. Specific targeting of Brn-3a may provide a useful strategy for regulating multiple tumor related genes involved with ovarian carcinomas.

## Introduction

Epithelial ovarian cancer is the fourth major cause of cancer morbidity and mortality in women. In spite of recent advances, the prognosis for a woman diagnosed with advanced-stage ovarian cancer has changed little over the last thirty years with a five-year survival of only 30% [1,2]. The majority of patients are diagnosed with Stage 3 or 4 disease, when the cancer has spread from the pelvis to the peritoneal cavity and the surrounding organs [2]. Under these circumstances aggressive local tumor growth involving invasion and metastasis occurs which often makes complete surgical removal of the cancer difficult. The causes of ovarian cancer and factors that influence the progression of the disease are only partially understood. A number of genetic abnormalities that have diagnostic and prognostic value have been determined [3,4], and some of the transcriptional and translational changes that contribute to the development and/or progression of the disease have been described [2], yet the underlying molecular pathways which initiate and regulate tumor progression still remain unknown. In contrast to almost all other cancers, ovarian cancer typically does not spread through the bloodstream. Instead, tumor growth is often limited to the abdominal (peritoneal) cavity, even in advanced cases. In advanced-stage patients cancer cells from the surface of the tumors are shed into the abdomen where they circulate in ascites (tumor fluid) as cellular aggregates and attach at different sites within the abdomen [5,6]. Debulking surgery followed by six cycles of combination chemotherapy, consisting of cisplatin and paclitaxel, is successful in initiating remission in 70-80% patients but it fails to get rid of any residual microscopic disease. As a consequence, within few months these patients return with recurrent cancer[1]. In most cases, patients present themselves with multiple sites of metastatic disease within the abdomen which are not treatable by secondary surgical removal resulting in bad prognosis. Hence, better approaches are needed not only to treat the primary cancer but also to inhibit the growth of recurrent disease. This can be achieved through a better understanding of the alteration and expression of transcription factors that regulate cellular growth, differentiation and apoptosis.

Brn-3 transcription factors (Brn-3a, 3b, 3c) are POU proteins (pit, Oct, Unc) and belong to the class IV homeobox family [7,8]. These transcription factors were identified originally in the nervous system [9,10], but are also expressed in reproductive tract tissues (breast, ovary, cervix, prostate, testis etc) [11]. They control the balance between cell proliferation, differentiation and apoptosis by targeting specific gene promoters either directly or through interactions with other cofactors [10,12]. Expression of these transcription factors has been reported to be altered in a number of different cancers. Brn-3a levels are significantly enhanced in cervical cancer [13,14], prostate cancer [15], neuroendocrine tumors [16] and Ewing's sarcoma [17]. On the other hand, Brn-3b expression is elevated in neuroblastomas [9,18] and in a subset of breast cancers [19,20] while Brn-3c expression is present in small cell carcinomas of the skin with poor prognosis [21].

The Brn-3a protein is encoded by a single gene but its transcription is regulated by two distinct promoters [22]. Transcription of this gene from the upstream promoter is followed by splicing to remove an intron between the first and second exon resulting in the long form of Brn-3a [Brn-3a(l)]. However, the use of a promoter within the intron downstream of the first exon, results in the formation of an un-spliced RNA encoding the short form of Brn-3a [Brn-3a(s)] that lacks the first 84 amino acids [10]. In some cases both forms of the proteins are produced in different proportion in different cells and they have different functional properties [10]. For example, Brn-3a(l) is over expressed in differentiating primary neurons and neuronal cell lines that are protected from stimuli that would generally induce apoptosis. This happens through activation and increased expression of anti-apoptosis genes, including Bcl-2 [10]. On the other hand, the ability to activate the promoters of differentiation-associated neurofilaments and neuronal outgrowth is dependent upon the C-terminal POU domain of Brn-3a and on both long and short forms of the molecule [10]. Thus, Brn-3a short and long have distinct functions in neuronal cells, Brn-3a(l) induces Bcl-2 expression and protects neurons from apoptosis, whereas, Brn-3a(s) induces the expression of differentiation-associated genes and induces neuronal differentiation [10]. Moreover, Brn-3 targets many other genes, particularly those with oncogeneic (such as ras and src) and apoptotic/anti-apoptotic roles (such as p53, Bcl-2, Bcl-x, Bax, p21, Hsp27) [18,20,23-26]. A recent review hypothesizes an oncogeneic role of Brn-3a by linking it with Bcl-2/VEGF induction involved in tumor angiogenesis [27], further implicating the role of this neuronal transcription factor in tumor progression.

In view of the evidence for the expression of Brn-3a transcription factor in non-neuronal cancer cell types of reproductive origin, we investigated the expression of Brn-3a(l) in normal ovaries and in different histological grades of ovarian carcinomas by immunohistochemistry. We also investigated the expression of Brn-3a(l) in ascites tumor cells and ovarian cancer cell lines by Western blot. The difference in the expression of Brn-3a was also evaluated in normal ovarian and cancer cell lines by immunofluorescence. We report distinct expression pattern of Brn-3a(l) in primary tumors, ascites tumor cells and ovarian cancer cell lines consistent with novel distinct role of this factor in the progression and recurrence of this disease.

## Methods and materials

### Antibodies and reagents

Mouse monoclonal and rabbit polyclonal Brn-3a antibodies were obtained from Santa Cruz Biotechnology Inc (Santa Cruz, CA, USA) and Millipore (Chemicon, Temecula, USA). The secondary antibodies and immunoperoxidase secondary detection system were purchased from Millipore (Chemicon, Temecula, CA, USA) and Invitrogen Corporation (Invitrogen, CA, USA). Western blotting detection reagents and analysis system were supplied by Amersham Biosciences (Amersham, UK).

### Cell lines

The human epithelial ovarian cancer lines OVCA 433, OVCA 429 and SKOV3, obtained from Dr Robert Bast, MD Anderson Centre, Houston, USA were described previously [28,29]. Ovarian cancer cell line 2008 was obtained from Dr Izi Haviv, Peter McCallum Cancer Centre, Melbourne, Australia. Non-tumorgenic SV40 antigen immortilized human ovarian surface epithelium derived cell lines (IOSE29 and IOSE80) has been described previously [30,31], were obtained from Dr Nelly Auersperg, University of British Columbia, Canada. These cell lines can be maintained in culture for several passages. IOSE29 and IOSE80 cell lines are not tumorigenic in mouse and mimic normal ovarian cells in culture. Cell lines were grown as monolayers in 25 cm^2 ^or 75 cm^2 ^flasks (Nunclon, Roskilde, Denmark) in complete growth medium consisting of 50% medium 199 (Sigma-Aldrich, Sydney, Australia) and 50% MCDB131 (Sigma-Aldrich, Sydney, Australia) supplemented with 10% (v/v) heat inactivated FBS and 2 mM glutamine (Invitrogen Corporation, CA, USA) in the presence of 37°C with 5% CO_2_.

### Tissues

This study was approved by the Research and Human Ethics Committee (HEC # 09/09) of The Royal Women's Hospital, Melbourne, Australia. The subjects were recruited after the provision of a participant information statement and with informed consent. Ovarian cancer patients with serous, mucinous, endometrioid, clear cell carcinoma and mixed subtypes were included in the study. The histopathological diagnosis and tumor grades were determined independently by staff pathologists. Histological grading was assigned as described by Silverberg [32]. Non-cancerous ovarian tissues were obtained from patients undergoing surgery as a result of suspicious ultrasound images, palpable abdominal masses and/or a family history of ovarian cancer. Description of patients who participated in the study is provided in Additional file 1 (Table 1).

### Preparation of tumor cells from ascites of ovarian cancer patients

100-500 ml of ascites was collected from patients diagnosed with advanced-stage serous ovarian carcinomas. Ascites was centrifuged and the contaminating red blood cells were removed by giving the cell suspension a hypotonic shock for 1 minute in sterile MilliQ H_2_O. The remaining cells were re-suspended in growth medium and counted using the Trypan Blue exclusion method. Initially some lymphocytes and fibroblasts were present but were easy to distinguish. Lymphocytes were small, smooth and perfectly round cells. Fibroblasts were long elongated cells whereas tumor cells were large with visible nuclei. In many cases, large multinucleated tumor cells were visible. Tumor cell cultures were incubated at 37°C in 5% CO_2 _in growth medium containing 50% Dulbecco Modified Eagle's Medium (DMEM) (Sigma-Aldrich, Sydney, Australia) and 50% MCDB131 (Invitrogen, CA, USA) supplemented with 10% (v/v) heat inactivated FBS and 2 mM glutamine (Invitrogen CA, USA). After 1-2 weeks, cultured cells were screened for the presence of tumor cells and contaminating fibroblasts by the cell surface expression of fibroblast surface protein (FSP), CA-125 and EpCAM (Sapphire Bioscience, Melbourne, Australia) using a flow cytometer (Becton and Dickinson, USA). Initially the expression of FSP was detected in 50% of the cultures. Confluent culures were split at 1:2 and after 3-4 passages the cultures were screened again for FSP, CA-125 and EpCAM. Sustained expression of CA-125 and EpCAM was observed in 3-4 passage cultures with significantly low expression of FSP indicating the over riding dominance of epithelial tumor cells with very few contaminating cells expressing FSP.

### Immunohistochemistry

Immunohistochemical analysis of ovarian tissues was performed as described previously [33,34]. Briefly, paraffin sections were cut at 4 μm thickness, mounted on silane coated slides and incubated overnight at 37°C. Sections were washed with distilled water after two changes of xylene and three changes of ethanol. Antigen retrieval was performed using citrate buffer (pH 6.0) and sections were held in Tris buffered saline (TBS). Endogenous peroxidase activity was removed using 3% hydrogen peroxide in methanol. The sections were incubated for 1 h in primary antibody (mouse monoclonal Brn-3a antibody, Santa Cruz, CA, USA) diluted 1/200 in 1% BSA in Tris buffer (100 mM, pH 7.6) at room temperature. Antibody binding was amplified using biotin and streptavidin HRP (Chemicon, CA, USA) for 15 min each and the complex was visualized using diaminobenzidine (DAB). Nuclei were lightly stained with Mayer's haematoxylin. Control IgG was used as a negative control.

Sections were assessed microscopically for positive DAB staining. Two observers independently evaluated the immunostaining results. The concordance ratio was >95%. Four sections were assessed per tissue sample and the subcellular distribution of staining was determined. Parallel sections were stained with hematoxylin and eosin to confirm the pathology diagnosis.

### Interpretation of staining results

The staining pattern of Brn-3a was evaluated as follows:

1. Immunoreactive Brn-3a was localized in the cytoplasm and/or nucleus of epithelial and stromal cells;

2. The extent of positive staining was deduced using the following scale: for each specimen, the positive staining extent was scored in 5 grades, namely, 0 (≤10%), 1 (≥11-25%), 2 (≥26-50%), 3 (≥51-75%), 4 (≥76-90%) and 5 (≥90~100%). The intensity of staining was further classified as low, moderate and high according to the intensity of DAB staining.

### Immunofluorescence

Immunofluorescence analysis of Brn-3a was performed by using the rabbit polyclonal Brn-3a antibody (Chemicon, Temecula, USA) as described previously [29]. Mouse monoclonal anti-mouse β-actin (Sigma, Melbourne, Australia) was used as an internal control. Alexa Fluor^® ^488 (goat anti-mouse IgG) and Alexa Fluor^® ^555 (goat anti-rabbit IgG) (Invitrogen, Melbourne Australia) were used as secondary antibodies. Images were visualized and captured by the fluorescence microscope (Olympus AX-70, Olympus, Australia), photographed and analysed with Zeiss AxioCam Axiovision software (Carl Zeiss Inc., New York, USA).

### SDS-PAGE and Western blot analysis

SDS-PAGE and Western blot was performed on cell lysates as described previously [34]. Mouse monoclonal Brn-3a antibody (Santa Cruz, CA, USA) was used for the detection of the 43 kDa Brn-3a. Protein loading was monitored by stripping the membrane with Restore Western blot Stripping Buffer (Thermo Scientific, MA, USA) and re-probing the membrane with β-actin primary antibody (Sigma-Aldrich, Sydney, Australia).

### Statistical analysis

Statistical analysis of the extent of Brn-3a(l) immunostaining between normal and tumor groups was determined by using Chi-squared test using the SPSS statistical software. In addition, the differences of the extent of staining between each individual tissue type (normal and different histological grades of tumors) were analyzed by non-parametric Kruskal Wallis test followed by Dunn's Multiple Comparison post tests. All data were considered significantly different from each other at p < 0.05.

## Results

### Immunohistochemical expression of Brn-3a in ovarian tissues

Mouse monoclonal Brn-3a antibody (Santa Cruz, CA, USA) was used for immunohistochemical analysis. This antibody was raised against amino acids 1-109 of Brn-3a of mouse origin and is specific for mouse, rat and human Brn-3a(l) form. Some ovarian tissue sections displayed some degree of background staining in the stroma possibly due to cross reaction of stromal factors with the Brn-3a antibody.

### Non-cancerous (normal) ovarian tissues

**Figure 1:**
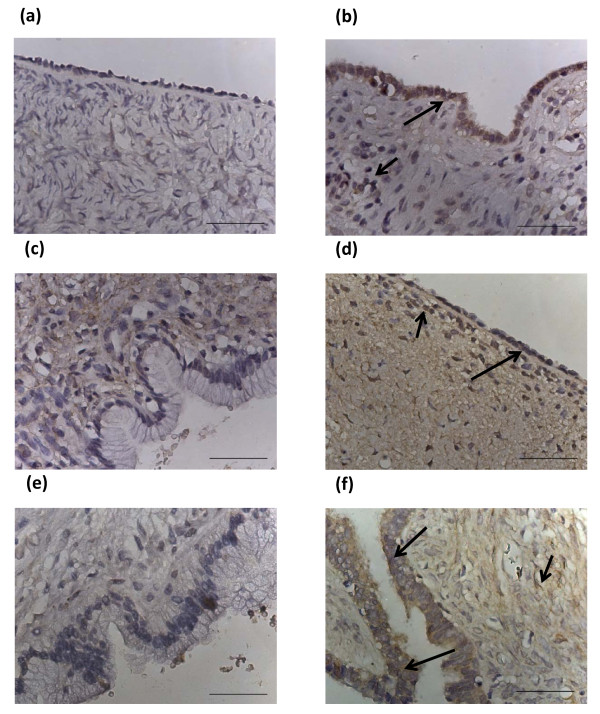
**Expression of Brn-3a(l) in normal ovaries, benign and borderline tumors**. Archival ovarian tissues were stained by the method described in the Materials and Methods. (a) Normal ovary, no epithelial or stromal staining; (b) normal ovary, moderate epithelial staining indicated by a long arrow; (c) benign mucinous tumor, no epithelial or stromal staining; (d) benign serous ovarian tumor, positive for epithelial (long arrow) and stromal (short arrow) Brn-3a(l) staining; (e) borderline mucinous tumor negative for epithelial and stromal staining and (f) borderline serous ovarian tumor positive for epithelial (long arrows) and stromal (short arrow) staining. Magnification-400; scale = 50 μm.

Out of eight normal ovarian sections examined, seven displayed no Brn-3a(l) staining on the ovarian surface epithelial cells (Fig 1a) while one showed moderate staining (Fig 1b). Weak to moderate stromal staining was observed in three normal ovaries. Staining in the stroma was both nuclear and cytoplasmic. Hence, 12% of non-cancerous normal ovarian tissues displayed epithelial staining in contrast to 40% stromal staining.

### Benign and borderline tumors

Benign tumors exhibited similar epithelial staining as normal ovaries with six out of seven benign tumors exhibiting no staining while one demonstrated moderate nuclear and cytoplasmic staining (11%) (Figs 1c and 1d). In contrast, the associated stromal tissues of three benign tumor tissues exhibited some staining confined to both nucleus and cytoplasm. Hence, 11% of benign tumors demonstrated epithelial staining compared to 50% of stromal staining.

The pattern of staining in borderline ovarian tumors varied with three tumors exhibiting no staining with other three demonstrating weak and one moderate epithelial staining (Figs 1e and 1f). Weak stromal staining of five tumors was evident while one showed moderate staining. Both nuclear and cytoplasmic staining was observed in positive specimens. Overall compared to normal ovaries both benign and borderline tumors exhibited denser stromal staining. This was more prominently observed in tumors of mucinuous subtype. In short, 57% of borderline tumors exhibited weak to moderate Brn-3a(l) staining, whereas 86% of these exhibited similar stromal staining.

### Grades 1, 2 and 3 tumors

**Figure 2:**
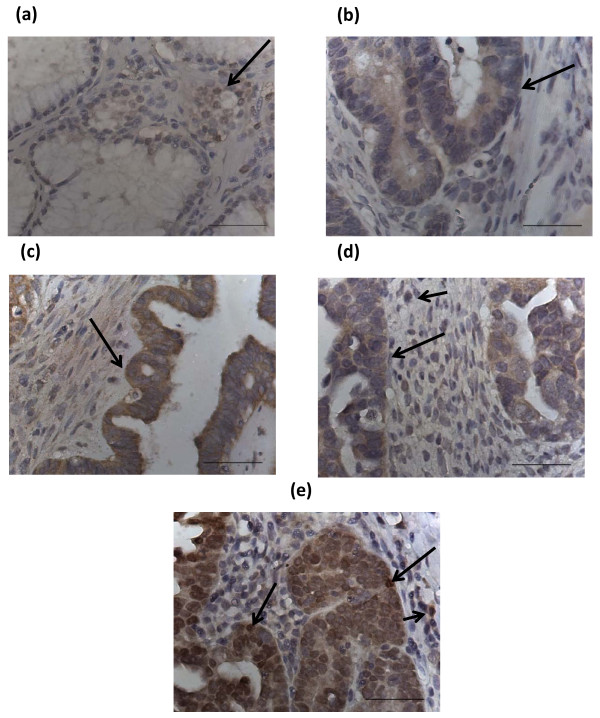
**Expression of Brn-3a(l) in grades 1, 2 and 3 ovarian tumors**. (a) Grade 1 mucinuous; (b) grade 1 endometriod; (c) grade 2 serous; (d) grade 3 serous and (e) grade 3 clear cell carcinoma tumors. Long arrows in each tumor illustrate positive Brn-3a(l) staining of the scattered epithelium. Short arrows in (d) and (e) indicate scattered stromal staining. Magnification-400; scale = 50 μm.

Grade 1 ovarian tumors exhibited more epithelial staining than their benign and borderline counterparts (Figs 2a &2b). 85% of grade 1 tumors exhibited weak to moderate Brn-3a(l) staining with six out of seven tumors demonstrating weak to moderate staining while one tumor did not show any staining at all. Staining of the epithelial cells was both nuclear and cytoplasmic. Weak to moderate stromal staining was also evident in 71% of samples. All grade 2 tumors exhibited epithelial Brn-3a(l) staining while stromal staining was evident in 87% of the samples (Fig 2c). 77% percentage of grade 3 patients demonstrated epithelial Bn-3a(l) staining compared to 88% stromal staining. Staining was confined to both nucleus and cytoplasm (Figs 2d and 2e). The staining intensity in both the epithelium and stroma of grades 1, 2 and 3 tumors was also enhanced.

**Figure 3:**
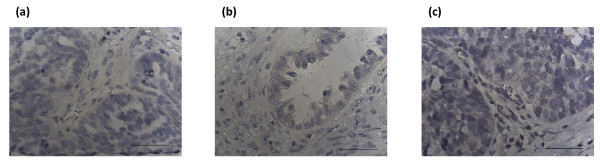
**Negative IgG controls for Brn-3a(l) staining in (a) grade 1 endometrioid (same tumor as in Fig 2b); (b) grade 2 serous (same tumor as in Fig 2c) and (c) grade 3 serous tumor (same tumor as in Fig 2d)**. Magnification-400; scale = 50 μm.

None of the tissues showed any positive staining with the control IgG (Figs 3a-c).

### Statistical analysis

**Figure 4:**
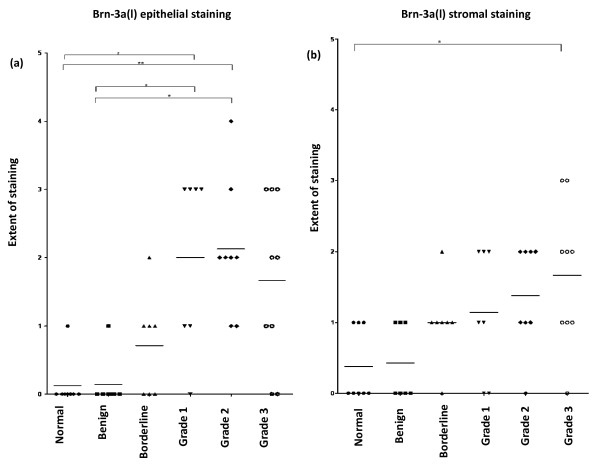
**Immunohistochemical expression of Brn-3a(l) in normal, benign, borderline, grade 1, grade 2 and grade 3 ovarian tumors**. Medians are shown as horizontal lines. Significant differences in the extent of epithelial and stromal Brn-3a(l) staining between normal and grades 1, 2 and 3 tumors are indicated by * (p < 0.01) and ** (p < 0.05).

By using Chi-squared test significant differences in the extent of Brn-3a epithelial as well as stromal immunostaining was determined between normal and histological grades of tumors (χ^2 ^= 41.01, df = 20, P = 0.004, χ^2 ^= 24.66, df = 15, P = 0.05). In parallel, the extent of Brn-3a staining was also significantly different in the epithelium of benign and borderline tumors compared to grades 1, 2 and 3 tumors (χ^2 ^= 14.33, df = 4, P = 0.006). To further analyze differences between each individual tissue and tumor types Kruskal Wallis and Dunn's Multiple Comparison post tests were performed. Compared to normal ovaries (0.13 ± 0.35, mean ± SD) and benign tumors (0.14 ± 0.38) significantly higher extent of epithelial Brn-3a(l) staining was observed in grades 1 (2.00 ± 1.29) and 2 tumors (2.13 ± 0.99) (p < 0.05) (Fig 4). In addition, stromal staining in grade 3 tumors (1.67 ± 1.00) was also significantly higher compared to normal ovaries (0.38 ± 0.52) (p < 0.05) (Fig 4).

### Brn-3a expression in normal ovarian and cancer cell lines as well as tumor cells isolated from ascites of advanced-stage cancer patients

#### Immunofluorescence analyses

**Figure 5:**
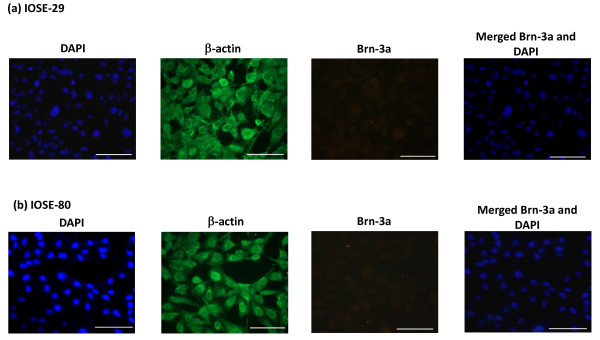
**Expression and localization of Brn-3a in (a-b) SV40 immortalised normal ovarian cell lines by fluorescent microscopy**. The expression and localization of Brn-3a was evaluated by using rabbit polyclonal Brn-3a antibody by fluorescent microscopy as described in the Methods and Materials. Cytoplasmic and nuclear staining were visualized using secondary Alexa 590 fluorescent labeled (red) antibody and DAPI (blue). Anti-mouse β-actin staining (green) followed by Alexa 488 (green) fluorescent labeling was used as an internal control. Magnification-400; scale = 50 μm.

**Figure 6:**
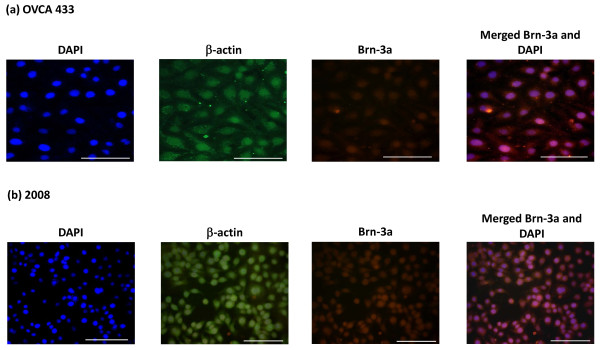
**Expression and localization of Brn-3a in (a-b) ovarian cancer cell lines by fluorescent microscopy**. The expression and localization of Brn-3a was evaluated by using rabbit polyclonal Brn-3a antibody by fluorescent microscopy as described in the Methods and Materials. Cytoplasmic and nuclear staining were visualized using secondary Alexa 590 fluorescent labeled (red) antibody and DAPI (blue). Anti-mouse β-actin staining (green) followed by Alexa 488 (green) fluorescent labeling was used as an internal control. Magnification-400; scale = 50 μm.

Immunofluorescence study was performed to determine the differences of Brn-3a expression in SV40 immortalized normal ovarian (IOSE29 and IOSE80) and cancer cell lines (OVCA433 and 2008) (Figs 5 and 6). As the Santa Cruz anti-Brn-3a(l) has the same mouse host as anti-β-actin (used as an internal control), the immunofluorescence experiment was performed with Millipore anti-rabbit Brn-3a antibody which recognizes both the short [Brn-3a(s)] and long forms of Brn-3a [Brn-3a(l)]. No significant Brn-3a expression was detected in immortalized normal ovarian cell lines (Figs 5a and 5b). On the other hand, positive cytoplasmic and nuclear Brn-3a staining was evident in both OVCA433 and 2008 ovarian cancer cell lines with 2008 demonstrating more staining than OVCA433 cell line (Figs 6a and 6b).

#### Western blot analyses

**Figure 7:**
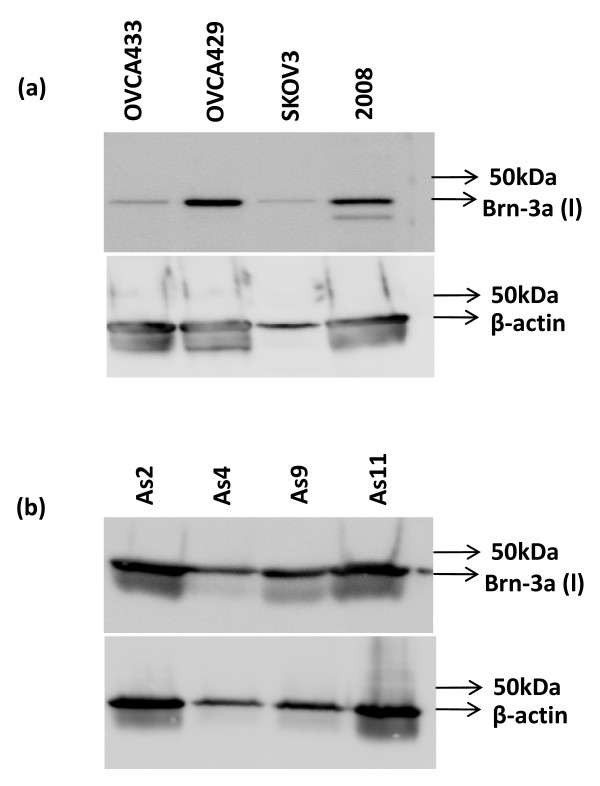
**Western blot analysis of Brn-3a(l) expression in (a) ovarian cancer cell lines and (b) tumor cells from ascites of cancer patients**. Cell lysates (25 μg) of (a) ovarian cancer cell lines (OVCA433, OVCA429, SKOV3 and 2008) and (b) tumor cells isolated from the ascites of patients with advanced-stage ovarian cancer (As2, As4, As9, As11) were prepared and loaded on 10% SDS-PAGE gels as described in the Methods and Material. Blots were probed with mouse monoclonal Brn-3a(l) antibody, stripped and re-probed with mouse monoclonal β-actin antibody as an internal protein loaded.

In order to determine the isoform expressed by ovarian cancer and tumor cells from ascites of ovarian cancer patients both Santa Cruz and Millipore antibodies were used. Western blotting was performed on cell lysates prepared from tumor cells isolated from patient's ascites and ovarian cancer cell lines OVCA433, OVCA429, 2008 and SKOV3. Santa Cruz anti-mouse Brn-3a antibody which recognizes the long form of Brn-3a demonstrated the expression of Brn-3a (l) (~43 kDa) in ovarian cancer cell lines and tumor cells isolated from patient's ascites (Figs 7a and 7b). The expression of Brn-3a(l) was relatively higher in OVCA429 and 2008 cells compared to OVCA433 and SKOV3 cells. This was consistent with immunofluorescence results which showed relatively higher expression of Brn-3a in 2008 cells compared to OVCA433 cell line (Figs 6a and 6b). Western blotting results using Santa Cruz anti-Brn-3a(l) antibody also demonstrated strong expression of Brn-3a(l) in the four ascites tumor samples which varied in expression with equal protein loading. Variable expression of β-actin which was used as an internal control was also demonstrated. In our hands we were unable to produce any Brn-3a band with Millipore anti-rabbit Brn-3a antibody indicating the unsuitability of this antibody for Western blot studies.

## Discussion

Malignant tumors initiate a transcriptional machinery to create a self-sustaining environment to break the neighboring cell barriers in order to facilitate migration and colonize to distant sites [35]. This is achieved by the acquisition, enhancement or alteration of the expression of transcription factors that initiate the transcriptional program needed for the metastatic process. These events are dependent on the over and under expression of molecules generally required for normal cellular functions. The indication that Brn-3a, a member of the Brn-3 family of type IV POU domain transcription factors is involved in the etiology of cancer has been demonstrated previously by the over expression of this transcription factor in CIN3 cervical lesions [13,14], neuroendocrine tumors [16], Ewing sarcomas [17] and prostate cancers [15]. Although the molecule is expressed at low levels in normal cervix and prostatic epithelium, it is significantly increased in CIN-3 lesions and prostate carcinomas. The expression of this transcription factor has been reported previously in normal ovaries [11] but not in ovarian carcinomas. In this study, we report enhanced expression of Brn-3a(l) in different histological grades and pathological subtypes of ovarian carcinomas as well as in the tumor cells isolated from ascites of ovarian cancer patients and in ovarian cancer cell lines.

Weak to moderate immunoreactivity of Brn-3a(l) was observed in almost all ovarian tumors studied. On the other hand, only 12% of normal ovaries had epithelial Brn-3a(l) immunoreactivity. The stromal staining even though relatively higher in the extent, constituted 40% of total staining. It should be noted that normal ovaries constituted of noncancerous ovarian tissues obtained from patients who have opted to remove their ovaries as a result of suspicious ultrasound images, palpable abdominal masses and/or a family history of ovarian cancer. Hence, it still remains to be determined if the observed Brn-3a(l) expression in one ovary is a consequence of genetic and/or clinical conditions of the patients involved or is a one off phenomenon of normal un-diseased ovary. This is consistent with an earlier study which reported weak expression of Brn-3a(l) in normal ovaries by Western blot [11]. Consistent with our results on normal ovaries, low expression of Brn-3a has also been reported in normal cervix and prostatic epithelium [14,15]. Weak expression of Brn-3a(l) was observed in benign ovarian tumors, while borderline ovarian tumors demonstrated weak to moderate cytoplasmic and nuclear expression. On the other hand, almost all ovarian tumors studied expressed Brn-3a(l) both in the epithelium as well as in the stroma. Enhanced expression of Brn-3a(l) in stromal cells of high grade tumors may contribute to the metastatic ability of tumors cells as demonstrated by the tumor growth enhancing effects of cancer associated fibroblasts [36] and infiltrating macrophages [37]. This is consistent with the previously described role of Brn-3a in tumorigenesis, and suggests its functionally active status in regulating the expression of key genes regulating tumor metastasis [13,15]. Consistent with the immunohistochemistry results, moderate to high cytoplasmic and nuclear expression of Brn-3a was observed in ovarian cancer cell lines. However, no Brn-3a expression was observed in immortalized normal ovarian cell lines, suggesting again that Brn-3a in ovarian neoplasms acts as an oncogene as demonstrated in case of cervical and prostate cancers [13,15].

Brn-3a(l) which possesses both the POU homeodomain and the N-terminal activation domain has been found to regulate the promoters of Bcl2 and p53 in human and mouse neurons [23-26]. Over expression of Brn-3a(l) in primary rat embryonic fibroblasts conferred on the cells a capacity for anchorage-independent cell growth [38]. In addition, the predominant expression of Brn-3a(l) in cervical cancers, neuroendocrine tumors and Ewing sarcomas has been reported previously [13,15,16]. Since ovarian cancer cells express Brn-3a(l), would suggest that this isoform of Brn-3a could have similar targets. Interestingly, the observation that Brn-3a(l) protects neurons from stimuli which would otherwise undergo apoptosis is consistent with a protective role of this isoform in ascites tumor cells exposed to an anchorage independent unfavorable microenvironment.

Taken together, our results indicate that Brn-3a may play an important role in the onset and progression of ovarian cancer. The neuroendocrine phenotype of ovarian tumors has been described previously [39]. Moreover, over expression of other neurotrophic factors such as brain derived neutrotrophic factor (BDNF) and neurotrophic tyrosine kinase receptor B (Trk B) have been demonstrated in high grade ovarian tumors, in metastatic ovarian lesions and in tumor cell aggregates of ascites [40]. However, none of these neurotrophic factors displayed any expression in normal epithelial ovarian tissues and benign ovarian tumors [40]. Our study demonstrates a similar expression profile of neurotrophic transcription factor Brn-3a in normal ovaries, benign tumors and different histological grades of ovarian tumors. Considering that Brn-3a is absent in 88% of normal ovaries and is elevated in ovarian carcinomas suggest that mutational changes in the ovaries may result in the overproduction of Brn-3a transcription factor which could facilitate the expression of neurotrophic factors [41] resulting in tumor progression, and anoikis suppression. The abnormal growth characteristics of ovarian tumors may thus be reversed by the reduction of endogenous Brn-3a(l) expression, making this factor an important target for therapeutic intervention.

## Competing interests

The authors declare that they have no competing interests.

## Authors' contributions

NA was involved with conceptualization, design, acquisition, analysis and interpretation of data, drafting and revising the manuscript. AL and CBR assisted with experiments, interpretation of data and manuscript preparation. JFK and MAQ assisted in the interpretation of data and edited the manuscript. All authors have read and approved the final manuscript.
